# Supplementary material for: Efficacy of Hip Strengthening on Pain Intensity, Disability, and Strength in Musculoskeletal Conditions of the Trunk and Lower Limbs: A Systematic Review with Meta-Analysis and Grade Recommendations
Source: Diagnostics (Basel). 2022 Nov 23;12(12):2910. doi: 10.3390/diagnostics12122910 (PMC9776732; doi:10.3390/diagnostics12122910)
Supplement: Supplementary file 1 [file diagnostics-12-02910-s001.zip › Supplementary S2 Search strategy.pdf]

Supplement File S2. Search strategy conducted on November 23rd 2018 and updated on March 31st 2020

OVID (MEDLINE, AMED, EMBASE, COCHRANE, PSYCINFO)

1. Randomized Controlled Trial/
2. Controlled Clinical Trial/
3. randomised controlled trial$.mp.
4. randomised controlled trial$.mp.
5. controlled clinical trial$.mp.
6. random allocation.mp. or Random Allocation/
7. Clinical Trial/
8. clinical trial$.mp.
9. Comparative Study/
10. cross-over studies.mp. or Cross-Over Studies/
11. Backache.mp. or Back Pain/
12. lumbago.mp. or Low Back Pain/
13. lumbar spine pain.mp.
14. (spin$ adj5 pain).mp.
15. aged.mp. [mp=ab, hw, kw, ti, ot, tx, ct, sh, tc, id, tm, tn, dm, mf, dv, fx, dq, nm, kf, px, rx, an, ui, sy]
16. aging.mp. [mp=ab, hw, kw, ti, ot, tx, ct, sh, tc, id, tm, tn, dm, mf, dv, fx, dq, nm, kf, px, rx, an, ui, sy]
17. older*.mp. [mp=ab, hw, kw, ti, ot, tx, ct, sh, tc, id, tm, tn, dm, mf, dv, fx, dq, nm, kf, px, rx, an, ui, sy]
18. elder*.mp. [mp=ab, hw, kw, ti, ot, tx, ct, sh, tc, id, tm, tn, dm, mf, dv, fx, dq, nm, kf, px, rx, an, ui, sy]
19. ancient*.mp. [mp=ab, hw, kw, ti, ot, tx, ct, sh, tc, id, tm, tn, dm, mf, dv, fx, dq, nm, kf, px, rx, an, ui, sy]
20. grey haired.mp. [mp=ab, hw, kw, ti, ot, tx, ct, sh, tc, id, tm, tn, dm, mf, dv, fx, dq, nm, kf, px, rx, an, ui, sy]
21. advanced in years.mp. [mp=ab, hw, kw, ti, ot, tx, ct, sh, tc, id, tm, tn, dm, mf, dv, fx, dq, nm, kf, px, rx, an, ui, sy]
22. 15 or 16 or 17 or 18 or 19 or 20 or 21 idade
23. 1 or 2 or 3 or 4 or 5 or 6 or 7 or 8 or 9 or 10 delineamento
24. 11 or 12 or 13 or 14 dor lombar (desfecho)
25. 22 and 23 and 24

PEDro

Abstract & Title: older* OR elder*

Therapy: not applicable

Problem: not applicable

Body Part: lumbar spine, sacro-iliac joint or pelvis

Subdiscipline: not applicable

Topic: not applicable

Method: clinical trial

Author/Association: not applicable

Title Only: not applicable

Source: not applicable

Published Since: not applicable

New records added since: not applicable

Score of at least: not applicable
