# Supplementary material for: Efficacy of Hip Strengthening on Pain Intensity, Disability, and Strength in Musculoskeletal Conditions of the Trunk and Lower Limbs: A Systematic Review with Meta-Analysis and Grade Recommendations
Source: Diagnostics (Basel). 2022 Nov 23;12(12):2910. doi: 10.3390/diagnostics12122910 (PMC9776732; doi:10.3390/diagnostics12122910)
Supplement: Supplementary file 1 [file diagnostics-12-02910-s001.zip › Supplementary S3 Reports excluded.pdf]

Supplement File S3. The detailed reports excluded			
	Author/Year of publication	Title	Reason for exclusion
1	Fiona Blackman & Elaine Atkins.(2014)	"The effect of adding grade B hip mobilization to a muscle strengthening home exercise programme on pain, function, and range of movement in adults with symptomatic early-stage hip osteoarthritis: A pilot study for a randomized controlled trial." International Musculoskeletal Medicine 36(2): 54-63.	Not comparison of interest
2	Bolgla., et al.(2015)	"Comparison of hip and knee strength in males with and without patellofemoral pain." Physical Therapy in Sport 16(3): 215-221.	Not-quasi RCT or RCT
3	Abbott, J. H., et al. (2013)	"Manual therapy, exercise therapy, or both, in addition to usual care, for osteoarthritis of the hip or knee: a randomized controlled trial 1: clinical effectiveness." Osteoarthritis and Cartilage 2013 Apr;21(4):525-534.	Not comparison of interest
4	Abbott, J. H., et al. (2009)	"Exercise therapy, manual therapy, or both, for osteoarthritis of the hip or knee: a factorial randomised controlled trial protocol." Trials 10: 11-11.	Not comparison of interest
5	Abbott, J. H., et al. (2019)	"Incremental clinical effectiveness and cost effectiveness of providing supervised physiotherapy in addition to usual medical care in patients with osteoarthritis of the hip or knee: 2-year results of the MOA randomised controlled trial." Osteoarthritis and Cartilage 2019 Mar;27(3):424-434.	Not comparison of interest
6	Ackland, D. C., et al. (2019)	"Hip abductor muscle volumes are smaller in individuals affected by patellofemoral joint osteoarthritis." Osteoarthritis and Cartilage 27(2): 266-272.	Not condition of interest
7	Actrn (2017)	"Targeted gluteal exercise program versus sham exercise program for people with hip osteoarthritis (the GHOST trial (Gluteal exercise for Hip OSTeoarthritis): a randomized controlled trial." http://www.who.int/trialsearch/Trial2.aspx?TrialID=ACTRN12617000970347.	Not condition of interest
8	Actrn (2018)	"Exercise program of the gluteal muscles and its effect on the vertical jump." http://www.who.int/trialsearch/Trial2.aspx?TrialID=ACTRN12618000751279.	Not condition of interest
9	Actrn (2019)	"Study of the effectiveness of conventional physiotherapy treatment supplemented with a protocol for strengthening the gluteus maximus in patients with femoral patellar syndrome." http://www.who.int/trialsearch/Trial2.aspx?TrialID=ACTRN12619001717145.	Not comparison of interest
10	Alenezi, M., et al. (2016)	"Effectiveness of movement imagery on hip abductors muscle strength: results from a randomised controlled trial and implications for musculoskeletal physiotherapy." Physiotherapy 102: e55-e56.	Not condition of interest
11	Alenezi, M. M., et al. (2016)	"Mental training approaches in physiotherapy: Exploring the role of imagery practice on hip abductors muscle strength--a randomised controlled trial...The 35th Scientific Meeting of the Physiotherapy Research Society 16 April 2016, University of Leicester, UK." International Journal of Therapy & Rehabilitation 23(6): S274-S274.	Not condition of interest
12	Andersen, V., et al. (2018)	"Electromyographic comparison of barbell deadlift, hex bar deadlift, and hip thrust exercises: a cross-over study." journal of strength & conditioning research 32(3): 587-593.	Not-quasi RCT or RCT
13	Aoyama, M., et al. (2019)	"A Prospective, Randomized, Controlled Trial Comparing Conservative Treatment With Trunk Stabilization Exercise to Standard Hip Muscle Exercise for Treating Femoroacetabular Impingement: A Pilot Study." Clinical Journal of Sport Medicine 29(4): 267-275.	Not condition of interest
14	Ashok, C. (2012)	Ashok, C. (2012). "Effects of Hip Abductor Muscle Strengthening Exercises in Patients with Osteoarthritic Knee Joints." Indian Journal of Physiotherapy & Occupational Therapy 6(4): 32-37.	Not condition of interest
15	Avraham, F., et al. (2007)	"The efficacy of treatment of different intervention programs for patellofemoral pain syndrome: a single blinded randomized clinical trial. Pilot study." The Scientific World Journal 2007 Aug 24;7:1256-1262.	Not condition of interest
16	Barbalho, M., et al. (2020)	"Back Squat vs. Hip Thrust Resistance-training Programs in Well-trained Women." International Journal of Sports Medicine 41(5): 306-310.	Not condition of interest

17	Barnes, R. Y., et al. (2020)	"Effect of a core stability, m. Gluteus medius and proprioceptive exercise programme on dynamic postural control in netball players." south african journal for research in sport, physical education & recreation 42(1): 1-11.	Not condition of interest
18	Beazley, D., et al. (2017).	"Trunk and hip muscle activation during yoga poses: Implications for physical therapy practice." Complementary Therapies in Clinical Practice 29: 130-135.	Not-quasi RCT or RCT
19	Bennell, K. L., et al. (2014).	"Effect of physical therapy on pain and function in patients with hip osteoarthritis: a randomized clinical trial." JAMA 2014 May 21;311(19):1987-1997.	Not comparison of interest
20	Bennell, K. L., et al. (2010).	"Efficacy of a multimodal physiotherapy treatment program for hip osteoarthritis: a randomised placebo-controlled trial protocol." BMC Musculoskeletal Disorders 11: 238-238.	Not condition of interest
22	Bieler, T., et al. (2018).	"Exercise induced effects on muscle function and range of motion in patients with hip osteoarthritis." Physiotherapy Research International 23(1): 1-1.	Not comparison of interest
23	Bishop, B. N., et al. (2018).	"Electromyographic analysis of gluteus maximus, gluteus medius, and tensor fascia latae during therapeutic exercises with and without elastic resistance." international journal of sports physical therapy 13(4): 668-675.	Not-quasi RCT or RCT
24	Bolgla, L. A., et al. (2018).	"Trunk and hip muscle activation during yoga poses: Do sex-differences exist?" Complementary Therapies in Clinical Practice 31: 256-261.	Not condition of interest
25	Bolgla, L. A., et al. (2006).	"Hip and knee strength, emg activity, and kinematics in subjects with patellofemoral pain syndrome. (abstract)." journal of orthopaedic & sports physical therapy 36(1): a67-a67.	Not condition of interest
26	Boren, K., et al. (2011).	"Electromyographic analysis of gluteus medius and gluteus maximus during rehabilitation exercises." International Journal of Sports Physical Therapy 6(3): 206-216.	Not-quasi RCT or RCT
27	Bossen, D., et al. (2013).	"Effectiveness of a web-based physical activity intervention in patients with knee and/or hip osteoarthritis: randomized controlled trial." Journal of Medical Internet Research 2013 Nov;15(11):e257.	Not condition of interest
28	Brown, T., et al. (2002).	"Hip abductor strength, sensorimotor function and lateral stepping responses in young and older women...Platform & poster presentations for CSM 2003." Neurology Report 26(4): 184-185.	Not condition of interest
29	Bruzek, R., et al. (1996).	"The importance of the gluteus maximus muscle in the therapy of functional disorders of the sacro-iliac joint. [German]." Manuelle Medizin 34(2): 42-45.	Not condition of interest
30	Burns, S. A., et al. (2018).	"Effectiveness of physical therapy interventions for low back pain targeting the low back only or low back plus hips: a randomized controlled trial protocol." Brazilian Journal of Physical Therapy / Revista Brasileira de Fisioterapia 22(5): 424-430.	Not comparison of interest
31	c, R. B. R.	"Benefits of functional training in women with knee pain." http://www.who.int/trialsearch/Trial2.aspx?TrialID=RBR 8c7267.	Not-quasi RCT or RCT
32	Casartelli, N. C., et al. (2019).	"Exercise Therapy for the Management of Femoroacetabular Impingement Syndrome: Preliminary Results of Clinical Responsiveness." Arthritis care & research 71(8): 1074-1083.	Not condition of interest
33	Ceballos-Laita, L., et al. (2019).	"Effects of dry needling in hip muscles in patients with hip osteoarthritis: a randomized controlled trial [with consumer summary]." Musculoskeletal Science & Practice 2019 Oct;43:76-82.	Not condition of interest
34	Chang, S. J., et al. (2007). "	Hip abductor exercise and lateral stability in older adults at risk of falls...Combined Sections Meeting 2008: section on geriatrics poster and platform presentations. February 6-9, 2008, Nashville, TN." Journal of Geriatric Physical Therapy 30(3): 146-147.	Not condition of interest
35	Chen, Y. Y., et al. (2008).	Chen, Y. Y., et al. (2008). "(Rehabilitative exercise prescription for osteoarthritic patients at different stages after needle knife therapy) [Chinese - simplified characters]." Zhongguo	Not condition of interest

		Zuzhi Gongcheng yu Linchuang Kangfu [Journal of Clinical Rehabilitative Tissue Engineering Research] 2008 Jun 10;12(24):4773-4776.	
36	Cichanowski, H. R., et al. (2007).	"Hip strength in collegiate female athletes with patellofemoral pain." Medicine & Science in Sports & Exercise 39(8): 1227-1232.	Not-quasi RCT or RCT
37	Clifford, C., et al. (2019).	"Isometric versus isotonic exercise for greater trochanteric pain syndrome: a randomised controlled pilot study [with consumer summary]." BMJ Open Sport & Exercise Medicine 2019 Sep 21;5(1):e000558.	Not condition of interest
38	Cochrane, T., et al. (2005).	"Randomised controlled trial of the cost-effectiveness of water-based therapy for lower limb osteoarthritis." Health Technology Assessment (Winchester, England) 2005 Aug;9(31):1-130.	Not condition of interest
39	Cowan, S. M., et al. (2009).	"Altered hip and trunk muscle function in individuals with patellofemoral pain." British Journal of Sports Medicine 43(8): 584-588.	Not comparison of interest
40	Ctri (2017).	"Effect of hip muscle strengthening on sacroiliac joint dysfunction."http://www.who.int/trialsearch/Trial2.aspx?TrialID=CTRI 04(008438).	Not condition of interest
41	Deyle, G. D., et al. (2000).	"Manual physical therapy and exercise improved function in osteoarthritis of the knee." Journal of Bone & Joint Surgery, American Volume 82(9): 1324	Not condition of interest
42	Deyle, G. D., et al. (2000).	"Effectiveness of manual physical therapy and exercise in osteoarthritis of the knee: a randomized, controlled trial." Annals of Internal Medicine 2000 Feb 1;132(3):173-181.	Not condition of interest
43	Dieterich, A., et al. (2015).	"Differentiation of gluteus medius and minimus activity in weight bearing and non-weight bearing exercises by M-mode ultrasound imaging." Manual Therapy 20(5): 715-722.	Not condition of interest
44	Dolak, K., et al. (2012).	"Hip-Strengthening Exercises Before Functional Exercises Reduced Pain in Women with Patellofemoral Pain Syndrome." Journal of Bone & Joint Surgery, American Volume 94(10): 940-940.	Not comparison of interest
45	Dolak, K. L., et al. (2011).	"Hip Strengthening Prior to Functional Exercises Reduces Pain Sooner Than Quadriceps Strengthening in Females With Patellofemoral Pain Syndrome: A Randomized Clinical Trial...[corrected] [published erratum appears in J ORTHOP SPORTS PHYS THER 2011 Sep; 41(9):700]." Journal of Orthopaedic & Sports Physical Therapy 41(8): 560-570.	Not comparison of interest
46	Earl, J. E. (2005).	"Gluteus medius activity during 3 variations of isometric single-leg stance." Journal of Sport Rehabilitation 14(1): 1-11.	Not condition of interest
47	Eitzen, I., et al. (2015)	"No effects of a 12-week supervised exercise therapy program on gait in patients with mild to moderate osteoarthritis: a secondary analysis of a randomized trial." Journal of Negative Results in Biomedicine 2015 Mar 5;14(5):Epub.	Not condition of interest
48	Elliott, C., et al. (2018).	"Systematic Review of the Addition of Hip Strengthening Exercises for Adults with Patellofemoral Pain Syndrome." Internet Journal of Allied Health Sciences & Practice 16(4): 1-13.	Not-quasi RCT or RCT
49	Eun Hyuk, K., et al. (2015).	"Effect of Hip Abduction Exercise with Manual Pelvic Fixation on Recruitment of Deep Trunk Muscles." American Journal of Physical Medicine & Rehabilitation 94(3): 201-210.	Not-quasi RCT or RCT
50	Ferber, R., et al. (2015).	"Strengthening of the hip and core versus knee muscles for the treatment of patellofemoral pain: a multicenter randomized controlled trial [with consumer summary]." Journal of Athletic Training 2015 Apr;50(4):366-377.	Not comparison of interest
51	Fernandes, L., et al. (2017).	"Supervised neuromuscular exercise prior to hip and knee replacement: 12-month clinical effect and cost-utility analysis alongside a randomised controlled trial." BMC Musculoskeletal Disorders 18: 1-11.	Not condition of interest
52	Fernandes, L., et al. (2010).	"Efficacy of patient education and supervised exercise versus patient education alone in patients with hip osteoarthritis: a single blind randomized clinical trial." Osteoarthritis and Cartilage 2010 Oct;18(10):1237-1243.	Not condition of interest

53	Fredericson, M., et al. (2000).	"Hip abductor weakness in distance runners with iliotibial band syndrome." Clinical Journal of Sport Medicine 10(3): 169-175.	Not condition of interest
54	Ganderton, C., et al. (2018).	"Gluteal loading versus sham exercises to improve pain and dysfunction in postmenopausal women with greater trochanteric pain syndrome: a randomized controlled trial." Journal of Women's Health 2018 Jun;27(6):815-829.	Not condition of interest
55	Gao, M. X., et al. (2006).	"(Evaluation of the efficacy of exercise program in the remission of low back pain in manage tank soldiers) [Chinese - simplified characters]." Zhongguo Linchuang Kangfu [Chinese Journal of Clinical Rehabilitation] 2006 Feb 25;10(8):32-34.	Not comparison of interest
56	Green, J., et al. (1993).	"Home exercises are as effective as outpatient hydrotherapy for osteoarthritis of the hip." British Journal of Rheumatology 1993 Sep;32(9):812-815.	Not condition of interest
57	Guimaraes, C. Q., et al. (2010).	"Electromyographic activity during active prone hip extension did not discriminate individuals with and without low back pain." Revista Brasileira de Fisioterapia 14(4): 351-357.	Not condition of interest
58	Gwon, A.-J., et al. (2020).	"Effects of integrating Neurac vibration into a side-lying bridge exercise on a sling in patients with chronic low back pain: a randomized controlled study. Philadelphia, Pennsylvania, Taylor & Francis Ltd. 36: 907-915.	Not condition of interest
59	Harris-Hayes, M., et al. (2014).	"Persons With Chronic Hip Joint Pain Exhibit Reduced Hip Muscle Strength." Journal of Orthopaedic & Sports Physical Therapy 44(11): 890-898.	Not condition of interest
60	Harris-Hayes, M., et al. (2016).	"Movement-Pattern Training to Improve Function in People With Chronic Hip Joint Pain: A Feasibility Randomized Clinical Trial." Journal of Orthopaedic & Sports Physical Therapy 46(6): 452-461.	Not condition of interest
61	Harris-Hayes, M., et al. (2018).	"Reduced Hip Adduction Is Associated With Improved Function After Movement-Pattern Training in Young People With Chronic Hip Joint Pain." Journal of Orthopaedic & Sports Physical Therapy 48(4): 316-324.	Not condition of interest
62	Hartley, C., et al. (2020).	"High-impact exercise increased femoral neck bone density with no adverse effects on imaging markers of knee osteoarthritis in postmenopausal women." Journal of Bone and Mineral Research 2020 Jan;35(1):53-63.	Not condition of interest
63	Hertel, J., et al. (2004).	"Combining isometric knee extension exercises with hip adduction or abduction does not increase quadriceps EMG activity." British Journal of Sports Medicine 38(2): 210-213.	Not condition of interest
64	Hewett, T. E., et al. (2017).	"Effectiveness of Neuromuscular Training Based on the Neuromuscular Risk Profile." American Journal of Sports Medicine 45(9): 2142-2147.	Not condition of interest
65	Hinman, R. S., et al. (2010).	Hinman, R. S., et al. (2010). "Hip muscle weakness in individuals with medial knee osteoarthritis." Arthritis care & research 62(8): 1190-1193.	Not condition of interest
66	Homan, K. J., et al. (2013).	"The influence of hip strength on gluteal activity and lower extremity kinematics." Journal of Electromyography and Kinesiology 23(2): 411-415.	Not condition of interest
67	Horsak, B., et al. (2019).	"Effects of a lower extremity exercise program on gait biomechanics and clinical outcomes in children and adolescents with obesity: A randomized controlled trial." Gait & Posture 70: 122-129.	Not condition of interest
68	Hott, A., et al. (2019).	"Patellofemoral pain: one year results of a randomized trial comparing hip exercise, knee exercise or free activity." Scandinavian Journal of Medicine & Science in Sports.	Not condition of interest
69	Howell, D. (2016).	"Letter to the Editor: 'Effect of stretching with and without muscle strengthening exercises for the foot and hip in patients with plantar fasciitis: A randomized controlled single-blind clinical trial'." Manual Therapy 23: e12-e12.	Not comparison of interest
70	Ince, G., et al. (2006).	"Effects of a multimodal exercise program for people with ankylosing spondylitis." Physical Therapy 2006 Jul;86(7):924-935.	Not condition of interest

71	Irct (2017).	"A comparative study of stabilization exercise focusing pelvic floor muscles training and routine physiotherapy on pain, disability and muscles thickness in females with low back pain following pregnancy." http://www.who.int/trialsearch/Trial2.aspx?TrialID=IRCT2017060125732N18.	Not condition of interest
72	Jafarnezhadgero, A., et al. (2019).	"Effects of corrective training on drop landing ground reaction force characteristics and lower limb kinematics in older adults with genu valgus: a randomized controlled trial." Journal of Aging and Physical Activity 2019 Feb;27(1):9-17.	Not condition of interest
73	Ji-hyun, L., et al. (2013).	"Effects of Different Hip Rotations on Gluteus Medius and Tensor Fasciae Latae Muscle Activity During Isometric Side-Lying Hip Abduction." Journal of Sport Rehabilitation 22(4): 301-307.	Not condition of interest
74	Jones, M., et al. (2007).	"The efficacy of exercise as an intervention to treat recurrent nonspecific low back pain in adolescents." Pediatric Exercise Science 2007 Aug;19(3):349-359.	Not condition of interest
75	Juhakoski, R., et al. (2011).	"A pragmatic randomized controlled study of the effectiveness and cost consequences of exercise therapy in hip osteoarthritis." Clinical Rehabilitation 25(4): 370-383.	Not condition of interest
76	Kamonseki, D. H., et al. (2016).	"Effect of stretching with and without muscle strengthening exercises for the foot and hip in patients with plantar fasciitis: A randomized controlled single-blind clinical trial." Manual Therapy 23: e13-e14.	Not comparison of interest
77	Kean, C. O., et al. (2011).	"Relationship between hip adduction moment, hip abductor strength and progression of knee osteoarthritis." Osteoarthritis and Cartilage 1): S49.	Not condition of interest
78	Keaveny, T. M., et al. (2017).	"Greater Gains in Spine and Hip Strength for Romosozumab Compared With Teriparatide in Postmenopausal Women With Low Bone Mass." Journal of Bone & Mineral Research 32(9): 1956-1962.	Not condition of interest
79	Kemmler, W., et al. (2020).	"Effects of High-Intensity Resistance Training on Osteopenia and Sarcopenia Parameters in Older Men with Osteosarcopenia—One-Year Results of the Randomized Controlled Franconian Osteopenia and Sarcopenia Trial (FrOST)." Journal of Bone & Mineral Research 35(9): 1634-1644.	Not condition of interest
80	Kemp, J. L., et al. (2015).	"A phase II trial for the efficacy of physiotherapy intervention for early-onset hip osteoarthritis: study protocol for a randomised controlled trial." Trials 16(1): 26-26.	Not condition of interest
81	Kotteeswaran, K., et al. (2017).	"Effectiveness of hip muscle strengthening with open and closed kinetic chain exercises in patellofemoral pain syndrome." Biomedicine (India) 37(4): 521-526.	Not comparison of interest
82	Krauss, I., et al. (2011).	"Efficacy of conservative treatment regimes for hip osteoarthritis--evaluation of the therapeutic exercise regime "Hip School": a protocol for a randomised, controlled trial." BMC Musculoskeletal Disorders 12(1): 270-270.	Not condition of interest
83	Kraus, I., et al. (2014).	"Exercise therapy in hip osteoarthritis -- a randomized controlled trial [with consumer summary]." Deutsches Arzteblatt International 2014 Sep 1;111(35-36):592-599.	Not comparison of interest
84	Krause, D. A., et al. (2009).	"Electromyographical analysis of the gluteus medius in weight-bearing exercises." Journal of Orthopaedic & Sports Physical Therapy 39(1): A57-A57.	Not condition of interest
85	Lee, S. P. and C. Powers (2009).	"Does diminished hip muscle strength influence medial-lateral dynamic stability in females with patellofemoral pain?" Journal of Orthopaedic & Sports Physical Therapy 39(1): S14-S14.	Not condition of interest
86	Lehecka, B. J., et al. (2019).	"The effects of gluteal squeezes compared to bilateral bridges on gluteal strength, power, endurance, and girth." PeerJ 7.	Not condition of interest
87	Leinonen, V., et al. (2000).	"Back and hip extensor activities during trunk flexion/extension: effects of low back pain and rehabilitation." Archives of Physical Medicine & Rehabilitation 81(1): 32-37.	Not condition of interest

88	Lifshitz, L. (2008).	"Chronic groin pain: hip strength and flexibility in professional soccer players." British Journal of Sports Medicine 42(6): 531-531.	Not condition of interest
89	Lim, H. J., et al. (2005).	"Effects of home-based daily exercise therapy on joint mobility, daily activity, pain, and depression in patients with ankylosing spondylitis." Rheumatology International 2005 Apr;25(3):225-229.	Not condition of interest
90	Lord, S. R., et al. (1996).	"Exercise effect of dynamic stability in older women: a randomized controlled trial." Archives of Physical Medicine & Rehabilitation 77(3): 232-236.	Not condition of interest
91	Lowry, C. D., et al. (2008).	"Management of patients with patellofemoral pain syndrome using a multimodal approach: a case series." Journal of Orthopaedic & Sports Physical Therapy 38(11): 691-702.	Not condition of interest
92	Macedo, C. S. G., et al. (2010).	"Efeito do isostretching na resistencia muscular de abdominais, gluteo maximo e extensores de tronco, incapacidade e dor em pacientes com lombalgia (The isostretching effect in the muscle strength of gluteus maximus, abdominal and the trunk extensor, incapacity and pain in patients with low back pain) [Portuguese]." Fisioterapia em Movimento [Physical Therapy in Movement] 2010 Jan-Mar;23(1):113-120.	Not comparison of interest
93	Marques, S., et al. (2014).	"Effectiveness of the strengthening of pelvic floor muscles, adductors of hip, gluteus maximus and gluteus medius in the treatment of stress urinary incontinence: blind randomized clinical trial - partial results (Abstract number 207)." Proceedings of the 44th Annual Meeting of the International Continence Society.	Not condition of interest
94	McCrory, J. L., et al. (2004).	"The effect of a single treatment of the ProtonicsTM system on biceps femoris and gluteus medius activation during gait and the lateral step up exercise." Gait and Posture 19(2): 148-153.	Not condition of interest
95	McQuade, K. J. and A. S. de Oliveira (2011).	"Effects of progressive resistance strength training on knee biomechanics during single leg step-up in persons with mild knee osteoarthritis." Clinical Biomechanics 26(7): 741-748.	Not condition of interest
96	Medeiros, M. J., et al. (1977).	"Influence of isometric exercise and passive stretch on hip joint motion." Physical Therapy 57(5): 518-523.	Not condition of interest
97	Mellor, R., et al. (2018).	"Education plus exercise versus corticosteroid injection use versus a wait and see approach on global outcome and pain from gluteal tendinopathy: prospective, single blinded, randomised clinical trial [with consumer summary]." BMJ 2018 May 2;361:k1662.	Not condition of interest
98	Mendis, M. D. and J. A. Hides (2016).	"Effect of motor control training on hip muscles in elite football players with and without low back pain [with consumer summary]." Journal of Science and Medicine in Sport 2016 Nov;19(11):866-871.	Not condition of interest
99	Mooney, V., et al. (2001).	"Exercise treatment for sacroiliac pain." Orthopedics 24(1): 29-32.	Not comparison of interest
100	Multanen, J., et al. (2017).	"Effect of progressive high-impact exercise on femoral neck structural strength in postmenopausal women with mild knee osteoarthritis: a 12-month RCT." Osteoporosis International 28(4): 1323-1333.	Not condition of interest
101	Murphy, S. L., et al. (2008).	"Effects of activity strategy training on pain and physical activity in older adults with knee or hip osteoarthritis: a pilot study." Arthritis Care & Research 2008 Oct 15;59(10):1480-1487.	Not condition of interest
102	Nakagawa, T. H., et al. (2012).	"Trunk, Pelvis, Hip, and Knee Kinematics, Hip Strength, and Gluteal Muscle Activation During a Single-Leg Squat in Males and Females With and Without Patellofemoral Pain Syndrome." Journal of Orthopaedic & Sports Physical Therapy 42(6): 491-501.	Not condition of interest
103	Narouei, S., et al. (2020).	"Effects of core stabilization exercises on thickness and activity of trunk and hip muscles in subjects with nonspecific chronic low back pain." Journal of Bodywork and Movement Therapies 24(4): 138-146.	Not condition of interest

104	Nct (2015).	"Hip Strengthening and Manual Therapy for Heel Pain Patients." https://clinicaltrials.gov/show/NCT02624635.	Not comparison of interest
105	Nct (2014).	"Effect of Hip Versus Spinal Joint Mobilization on Hip Muscle Strength." https://clinicaltrials.gov/show/NCT02700594.	Not condition of interest
106	Nct (2014).	"Hip Strengthening Versus Quadriceps Based Training for Patellofemoral Pain Syndrome." https://clinicaltrials.gov/show/NCT02114294.	Not condition of interest
107	Nct (2017).	"Effect of Two Strengthening Protocols for Lower Limbs in Patients With Patellofemoral Pain." https://clinicaltrials.gov/show/NCT03163290.	Not condition of interest
108	Nct (2018).	"Effectiveness of 'Functional Range Conditioning' in Treating Patients With Chronic Non-specific Low Back Pain." https://clinicaltrials.gov/show/NCT03456050.	Not condition of interest
109	Nct (2016).	"Effect of Strengthening the Hip Abductor in Patients With Knee Osteoarthritis: randomized Controlled Trial." https://clinicaltrials.gov/show/NCT02901964.	Not comparison of interest
110	Olagbegi, O. M., et al. (2016).	"Effectiveness of combined chain exercises on pain and function in patients with knee osteoarthritis." Bangladesh Journal of Medical Science 2016 Apr;15(2):178-188.	Not condition of interest
111	Oliver, G., et al. (2012).	"The Effects of a Lumbopelvic-Hip Strengthening Intervention Program on Functional Testing in Collegiate Female Tennis Players." Clinical Kinesiology (Online Edition) 66(1): 13-18.	Not condition of interest
112	Palmer, K., et al. (2015).	"A randomised trial into the effect of an isolated hip abductor strengthening programme and a functional motor control programme on knee kinematics and hip muscle strength." BMC Musculoskeletal Disorders 16: 105.	Not condition of interest
113	Pinto, D., et al. (2013).	"Manual therapy, exercise therapy, or both, in addition to usual care, for osteoarthritis of the hip or knee. 2: economic evaluation alongside a randomized controlled trial." Osteoarthritis and Cartilage 2013 Oct;21(10):1504-1513.	Not comparison of interest
114	Pisters, M. F., et al. (2010).	"Long-term effectiveness of exercise therapy in patients with osteoarthritis of the hip or knee: a randomized controlled trial comparing two different physical therapy interventions." Osteoarthritis and Cartilage 2010 Aug;18(8):1019-1026.	Not comparison of interest
116	Rabelo, N. D. D. A. et al. (2014).	"Neuromuscular training and muscle strengthening in patients with patellofemoral pain syndrome: a protocol of randomized controlled trial." BMC Musculoskeletal Disorders 15(1): 157-157.	Not condition of interest
117	Rabelo, N. D. d. A., et al. (2017).	"Adding motor control training to muscle strengthening did not substantially improve the effects on clinical or kinematic outcomes in women with patellofemoral pain: A randomised controlled trial." Gait & Posture 58: 280-286.	Not condition of interest
118	Rathleff, M. S., et al. (2012).	"Early intervention for adolescents with patellofemoral pain syndrome--a pragmatic cluster randomised controlled trial." BMC Musculoskeletal Disorders 13(1): 9-9.	Not condition of interest
119	Rathnamala, D., et al. (2020).	"Effectiveness of gluteal and knee muscles strengthening in improving pain and disability in females with flexible flat foot." Drug Invention Today 13(6): 882-886.	Not comparison of interest
120	Ravaud, P., et al. (2004).	"Management of osteoarthritis (OA) with an unsupervised home based exercise programme and/or patient administered assessment tools. A cluster randomised controlled trial with a 2x2 factorial design." Annals of the Rheumatic Diseases 2004 Jun;63(6):703-708.	Not condition of interest
121	Rees, S. S., et al. (2008).	"Effects of whole-body vibration exercise on lower-extremity muscle strength and power in an older population: a randomized clinical trial." Physical Therapy 88(4): 462-470.	Not condition of interest
122	Regelski, C., et al. (2015).	"Hip strengthening compared with quadriceps strengthening in conservative treatment of patients with patellofemoral pain: A critically appraised topic." International Journal of Athletic Therapy & Training 20(1): 4-12.	Not comparison of interest

123	Reuteman, P. F. and T. Kernozek (2009).	"Strength differences of hip external rotators using 2 different methods of assessing isometric strength." Journal of Orthopaedic & Sports Physical Therapy 39(1): A46-47.	Not condition of interest
124	Rogan, S., et al. (2019).	"Effects of hip abductor muscles exercises on pain and function in patients with patellofemoral pain: a systematic review and meta-analysis." journal of strength & conditioning research 33(11): 3174-3187.	Not-quasi RCT or RCT
125	Saffari, M., et al. (2018).	"A theory of planned behavior-based intervention to improve quality of life in patients with knee/hip osteoarthritis: a randomized controlled trial." Clinical Rheumatology 2018 Sep;37(9):2505-2515.	Not condition of interest
126	Schoo, A. M., et al. (2005).	"The effects of mode of exercise instruction on compliance with a home exercise program in older adults with osteoarthritis [with consumer summary]." Physiotherapy 2005 Jun;91(2):79-86.	Not condition of interest
127	scwz, R. B. R. (2019).	"Investigation of the effectiveness of elastic taping associated with thigh and hip strengthening in the treatment of anterior knee pain." http://www.who.int/trialsearch/Trial2.aspx?TrialID=RBR 8s6cwz.	Not condition of interest
128	Sheerin, K. R., et al. (2012).	"Effects of a lower limb functional exercise programme aimed at minimising knee valgus angle on running kinematics in youth athletes." Physical Therapy in Sport 13(4): 250-254.	Not condition of interest
129	Shrier, I., et al. (2008).	"The feasibility of a randomized trial using a progressive exercise program in patients with severe hip osteoarthritis." Journal of Musculoskeletal Pain 16(4): 309-317.	Not condition of interest
130	Silva, N. C., et al. (2019).	"Effects of neuromuscular training and strengthening of trunk and lower limbs muscles in women with Patellofemoral Pain: A protocol of randomized controlled clinical trial, blinded." Trials 20(1): 586-586.	Not condition of interest
131	Sled, E. A., et al. (2010).	"Effect of a Home Program of Hip Abductor Exercises on Knee Joint Loading, Strength, Function, and Pain in People With Knee Osteoarthritis: A Clinical Trial." Physical Therapy 90(6): 895-904.	Not condition of interest
132	Soleimani, F., et al. (2017).	"Effectiveness of knee exercises versus combined knee and hip exercises in treatment of patellofemoral pain: a randomized clinical trial." Koomesh 19(3): 554-564.	Not comparison of interest
133	Stearns-Reider, K. M. and C. M. Powers (2018).	"Rate of Torque Development and Feedforward Control of the Hip and Knee Extensors: Gender Differences." Journal of Motor Behavior 50(3): 321-329.	Not condition of interest
134	Steinhilber, B., et al. (2014).	"Stiffness, Pain, and Hip Muscle Strength Are Factors Associated With Self-reported Physical Disability in Hip Osteoarthritis." Journal of Geriatric Physical Therapy 37(3): 99-105.	Not condition of interest
135	Storheim, K. and K. Bø (2000).	"The effect of intensive group exercise in patients with chronic low back pain." Advances in Physiotherapy 2(3): 113-123.	Not condition of interest
136	Stowers, T., et al. (1983).	"The short-term effects of three different strength-power training methods." National Strength & Conditioning Association Journal 5(3): 24-27.	Not condition of interest
137	Struminger, A. H., et al. (2013).	"Comparison of gluteal and hamstring activation during five commonly used plyometric exercises." Clinical Biomechanics 28(7): 783-789.	Not condition of interest
138	Sugimoto, D. A. I., et al. (2014).	"Effects of compliance on trunk and hip integrative neuromuscular training on hip abductor strength in female athletes." Journal of Strength & Conditioning Research 28(5): 1187-1194.	Not condition of interest
139	Svege, I., et al. (2015).	"Exercise therapy may postpone total hip replacement surgery in patients with hip osteoarthritis: a long-term follow-up of a randomised trial." Annals of the Rheumatic Diseases 2015 Jan;74(1):164-169.	Not condition of interest
140	Tan, S. S., et al. (2016).	"Cost-utility of exercise therapy in patients with hip osteoarthritis in primary care." Osteoarthritis & Cartilage 24(4): 581-588.	Not condition of interest

141	Tao, H. (2002).	"The research of the effect of hip stress lower-limbs strength training methods on dash technique and key specialized strength." Hong Kong Journal of Sports Medicine & Sports Science 15: 55-62.	Not condition of interest
142	Teirlinck, C. H., et al. (2016).	"Effectiveness of exercise therapy added to general practitioner care in patients with hip osteoarthritis: a pragmatic randomized controlled trial." Osteoarthritis and Cartilage 2016 Jan;24(1):82-90.	Not condition of interest
143	Thorborg, K., et al. (2014).	"Eccentric and Isometric Hip Adduction Strength in Male Soccer Players With and Without Adductor-Related Groin Pain." Orthopaedic journal of sports medicine: 1-7.	Not condition of interest
144	Thorborg, K., et al. (2016).	"Large strengthening effect of a hip-flexor training programme: a randomized controlled trial." Knee Surgery, Sports Traumatology, Arthroscopy 24(7): 2346-2352.	Not condition of interest
145	van Baar, M. E. (1999).	"(Effectiveness of exercise therapy for osteoarthritis of the hip or knee) [Dutch]." Nederlands Tijdschrift voor Fysiotherapie [Dutch Journal of Physical Therapy] 1999 May;109:2-5.	Not condition of interest
146	Wang, J., et al. (2020).	"Hip abductor strength–based exercise therapy in treating women with moderate-to-severe knee osteoarthritis: a randomized controlled trial." Clinical Rehabilitation 34(2): 160-169.	Not comparison of interest
147	Webster, K. and P. Gribble (2013).	"A comparison of electromyography of gluteus medius and maximus in subjects with and without chronic ankle instability during two functional exercises." Physical Therapy in Sport 14(1): 17-22.	Not condition of interest
148	White, S. H., et al. (2017).	"Submaximal exercise training improves mitochondrial efficiency in the gluteus medius but not in the triceps brachii of young equine athletes." Scientific reports 7(1): 14389-.	Not condition of interest
149	Wood, K., et al. (2020).	"The effects of closed and open kinetic chain exercise on strength and onset timing of hip musculature and dynamic knee valgus in women." Gazzetta Medica Italiana Archivio per le Scienze Mediche 179(4): 213-223.	Not comparison of interest
150	Yañez-Álvarez, A., et al. (2020).	"Effects of exercise combined with whole body vibration in patients with patellofemoral pain syndrome: a randomised-controlled clinical trial." BMC Musculoskeletal Disorders 21(1): N.PAG-N.PAG.	Not condition of interest
151	zdp, R. B. R. (2018).	"Effect of physiotherapeutical treatment on hip pain in women." http://www.who.int/trialsearch/Trial2.aspx?TrialID=RBR 84zdp6.	Not condition of interest
152	Saki, F., et al. (2022).	"The effects of gluteus medius and tibialis anterior kinesio taping on postural control, knee kinematics, and knee proprioception in female athletes with dynamic knee valgus." Physical Therapy in Sport 53: 84-90. Texto completo	Not condition of interest
153	DeJong Lempke, A. F., et al. (2022).	"Gluteal Activity During Gait in Patients With Chronic Ankle Instability Following Rehabilitation: A Randomized Controlled Trial." Journal of Sport Rehabilitation 31(2): 158-164.	Not condition of interest
154	Zaworski, K., et al. (2021).	"The effect of motor control training according to the Kinetic Control concept on the back pain of female football players." Journal of Back and Musculoskeletal Rehabilitation 34(5): 757-765.	Not condition of interest
155	Wu, Z., et al. (2021).	"Effects of whole-body vibration plus hip-knee muscle strengthening training on adult patellofemoral pain syndrome: a randomized controlled trial." Disability and rehabilitation: 1-9.	Not comparison of interest
156	Teschler, M., et al. (2021).	"Four weeks of electromyostimulation improves muscle function and strength in sarcopenic patients: a three-arm parallel randomized trial." Journal of Cachexia, Sarcopenia and Muscle 12(4): 843-854	Not condition of interest
157	Schwartz, A. M., et al. (2021).	"Capsular Management in Direct Anterior Total Hip Arthroplasty: A Randomized, Single-Blind, Controlled Trial." Journal of Arthroplasty 36(8): 2836-2842.	Not condition of interest
158	Scafoglieri, A., et al. (2021).	"Effectiveness of local exercise therapy versus spinal manual therapy in patients with patellofemoral pain syndrome: medium term follow-up results of a randomized controlled trial." BMC Musculoskeletal Disorders 2021 May 15;22(446):Epub	Not condition of interest
159	Sanika, V., et al. (2021).	"Comparison of Glutues Maximus Activation to Flexion Bias Exercises Along with MET Technique in Subjects with Anterior Rotated Sacroiliac Joint Dysfunction-a Randomised Controlled Trial." International Journal of Therapeutic Massage & Bodywork 14(1): 30-38	Not condition of interest

160	Rykov, K., et al. (2021).	"MRI Assessment of Muscle Damage After the Posterolateral Versus Direct Anterior Approach for THA (Polada Trial). A Randomized Controlled Trial." Journal of Arthroplasty 36(9): 3248-3248.	Not condition of interest
161	Oh, S. J. and S. H. Lee (2021).	"Comparing durability of water- and land-based exercise benefits among older adults in South Korea: A randomized controlled trial with 1-year follow-up." Journal of Back & Musculoskeletal Rehabilitation 34(5): 745-755	Not condition of interest
162	Kistler-Fischbacher, M., et al. (2021).	"A comparison of bone-targeted exercise with and without antiresorptive bone medication to reduce indices of fracture risk in postmenopausal women with low bone mass: the MEDEX-OP randomized controlled trial." Journal of Bone and Mineral Research 2021 Sep;36(9):1680-1693	Not condition of interest
163	Kisacik, P., et al. (2021).	"Short foot exercises have additional effects on knee pain, foot biomechanics, and lower extremity muscle strength in patients with patellofemoral pain." Journal of Back and Musculoskeletal Rehabilitation 2021;34(6):1093-1104	Not condition of interest
